# Reduced Plasma Nonesterified Fatty Acid Levels and the Advent of an Acute Lung Injury in Mice after Intravenous or Enteral Oleic Acid Administration

**DOI:** 10.1155/2012/601032

**Published:** 2012-02-27

**Authors:** Cassiano Felippe Gonçalves de Albuquerque, Patrícia Burth, Mauricio Younes Ibrahim, Diogo Gomes Garcia, Patrícia Torres Bozza, Hugo Caire Castro Faria Neto, Mauro Velho Castro Faria

**Affiliations:** ^1^Laboratório de Imunofarmacologia, Fundação Oswaldo Cruz, FIOCRUZ, Rio de Janeiro 21040-900, Brazil; ^2^Departamento de Biologia Celular e Molecular, Instituto de Biologia, Universidade Federal Fluminense, Niteroi 24020-150, Brazil; ^3^Departamento de Medicina Interna, Faculdade de Ciências Medicas, Universidade do Estado do Rio de Janeiro, Rio de Janeiro 20550-900, Brazil

## Abstract

Although exerting valuable functions in living organisms, nonesterified fatty acids (NEFAs) can be toxic to cells. Increased blood concentration of oleic acid (OLA) and other fatty acids is detected in many pathological conditions. In sepsis and leptospirosis, high plasma levels of NEFA and low albumin concentrations are correlated to the disease severity. Surprisingly, 24 h after intravenous or intragastric administration of OLA, main NEFA levels (OLA inclusive) were dose dependently decreased. However, lung injury was detected in intravenously treated mice, and highest dose killed all mice. When administered by the enteral route, OLA was not toxic in any tested conditions. Results indicate that OLA has important regulatory properties on fatty acid metabolism, possibly lowering circulating fatty acid through activation of peroxisome proliferator-activated receptors. The significant reduction in blood NEFA levels detected after OLA enteral administration can contribute to the already known health benefits brought about by unsaturated-fatty-acid-enriched diets.

## 1. Introduction

 Nonesterified fatty acids (NEFAs) are transported by the blood stream bound to albumin, a condition avoiding their cytotoxicity [1, 2]. Besides being an important fuel for the energetic metabolism, they also modulate leukocyte function, acting as signaling molecules [3–5]. Several cell types exhibit morphological features of apoptosis and necrosis after NEFA exposure [6, 7]. Oleic (OLA) and linoleic acids activate caspases 3 and 6, enhancing the generation of reactive oxygen species and a significant mitochondrial depolarization in leukocytes [8, 9].

 Symptom severity in diseases as sepsis, leptospirosis, and pancreatitis is associated to increased serum NEFA [10–13]. Severe leptospirosis and sepsis are also characterized by a concomitant decrease in plasma albumin concentration consequent to a functional liver injury or *increased* vascular permeability possibly caused by NEFA toxicity [13–15]. Accordingly, increased OLA and decreased albumin plasma levels seem to predict the development of acute respiratory distress syndrome (ARDS) [16, 17]. Since OLA and other nonesterified unsaturated fatty acids are potent Na/K-ATPase inhibitors, whether *in vitro* [18, 19] or *in vivo* [20], the involvement of the lung Na/K pump inhibition in the advent of ARDS has to be considered. In experimental animals, intravenous OLA injection can induce acute lung injury (ALI) [21, 22]. This syndrome is characterized by neutrophil infiltration and edema formation [23], due to increased endothelial permeability and loss of epithelial barrier function [24], causing neutrophil and macrophage accumulation in the lung. Upon activation, these cells produce inflammatory mediators [25]. Lipid bodies (lipid-rich inclusions found in the leukocyte cytosol) are also augmented in ALI [26]. They act as amplifiers of inflammatory lipid mediator production such as prostaglandin E_2_ (PGE_2_) in macrophages and leukotriene B_4_ (LTB_4_) in macrophages and neutrophils [27]. In the present work, such parameters were used to characterize the onset of ALI after intravenous oleic acid administration.

On the other hand, many reports highlight the association of unsaturated fatty acid diets to a healthy lifestyle. The well-known Mediterranean diet contains large amounts of olive oil, which is rich in the esterified form of OLA [28]. Furthermore, dietary monounsaturated fatty acids were considered protective against metabolic syndrome and cardiovascular disease risks [29]. Populations using such diets have reduced serum triglycerides and lower incidence of cardiovascular problems [30, 31].

The present study aimed at a better understanding of some deleterious and putative beneficial effects of OLA, when directly administered to mice. We investigated the consequences of increasing OLA doses, administered by intravenous or intragastric routes, on plasma NEFA concentration and on the triggering of an acute lung injury.

## 2. Material and Methods

### 2.1. Animals

All experiments were conducted on male Swiss mice weighting 33 ± 3 g obtained from the Oswaldo Cruz Foundation breeding unit. Animals were lodged at 22°C with a 12 h light/dark cycle and free access to food and water. Animal housing conditions and all experimental procedures conformed to institutional regulations and were in accordance with the National Institute of Health guidelines on animal care. All procedures described here were approved by the Institutional Animal Welfare Committee under license number 002-08.

### 2.2. Preparation of Tris-Oleate Solutions

Oleic acid obtained from Sigma Chemicals was used to prepare a 100 mmol/L tris-oleate solution. After weighting and water addition, tris powder (Trisma base-Sigma) was slowly added until the pH reached 10.0. The mixture was sonicated for complete tris-oleate solubility and then the pH was carefully adjusted to 7.6 with diluted HCl. Working oleate solutions were prepared by appropriate dilutions of the 100 mmol/L solution with phosphate buffered saline (PBS) pH 7.6.

### 2.3. Intravenous Administration of Oleate

Intravenous injections were performed into the orbital plexus (inner angle of the eye ball), and blood was collected by cardiac puncture 24 h latter. In some experiments, blood samples were collected 6 after the injection. Control groups received 100 *μ*L of saline. Other groups received 100 *μ*L of tris-oleate solutions corresponding to oleate doses of 20, 40, 80, and 160 mg/kg.

### 2.4. Intragastric Administration of Oleate

 A thin catheter coupled to a 1.0 mL syringe was introduced through the mouse esophagus and 100 *μ*L of the appropriate oleate solution or PBS (control animals) were injected into the gastric lumen. Oleate doses of 20, 40, 80, and 160 mg/kg were also used. Blood was collected by cardiac puncture 24 h latter.

### 2.5. Plasma NEFA Quantification

Plasma concentrations of the predominant NEFA—palmitic, oleic, linoleic, palmitoleic, and stearic acids—were determined by high performance liquid chromatography (HPLC) as described by Puttman et al. [32]. Methodological details were delineated in a previous publication [13].

### 2.6. Albumin Quantification

 Plasma albumin concentration was determined by the colorimetric procedure of Doumas et al. [33] using bovine serum albumin solutions as standards.

### 2.7. Total and Differential Cell Analysis on Bronchoalveolar Lavage Fluid (BALF)

The bronchoalveolar lavage was performed after isolating the trachea by blunt dissection. A small caliber tube was inserted and secured in the airway. PBS (1.0 mL) was then instilled and gently aspirated. This procedure was repeated three times, and collected fluids were pooled. In every instillation/aspiration cycle, the same volume (1.0 mL) was recovered from each animal. Total leukocyte counts were performed by light microscopy in Neubauer chambers after diluting BALF samples in Türk's solution (2% acetic acid). Differential leukocyte counts were determined in cytocentrifuged smears stained by the May-Grunwald-Giemsa method. Total BALF protein was determined by the Micron BCA Kit method (Pierce) according to the manufacturer's instructions.

### 2.8. Lipid Body Staining and Counting

 While still moist, leukocytes on cytospin slides were fixed in 3.7% formaldehyde in Ca^2+^, Mg^2+^-free Hank's balanced salt solution (HBSS), pH 7.4 and stained with 1.5% OsO_4_ as described in Bozza et al. [34]. Lipid bodies were counted by light microscopy with oil immersion objective lens in 50 consecutively scanned leukocytes.

### 2.9. PGE_2_ and LTB_4_ Assays

LTB_4_ and PGE_2_ in BALF supernatants were assayed by ELISA kits according to the manufacturer's instructions (Cayman Chemical, Ann Arbor, MI).

### 2.10. Statistical Analysis

Results were expressed as mean ± SEM and were analyzed by the Neuman-Keuls-Student test. Differences were considered significant when *P* < 0.05.

## 3. Results

**Figure 1:**
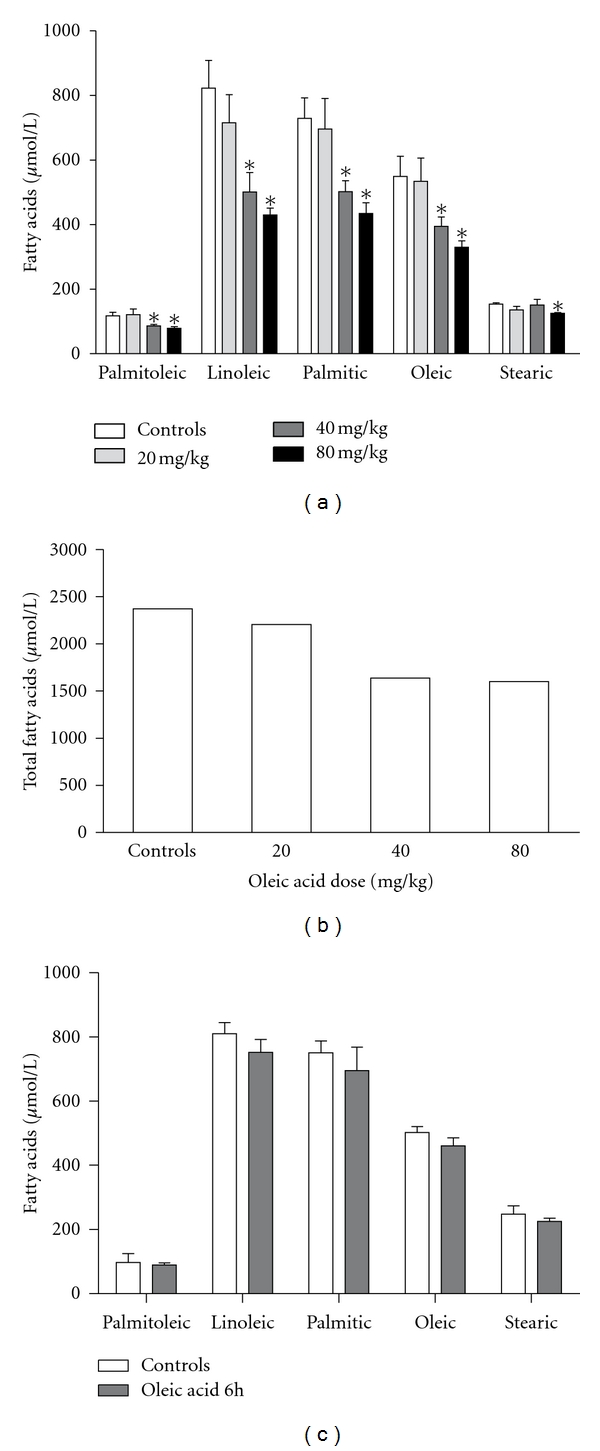
Plasma NEFA concentrations in mice after intravenous OLA injections. (a) Plasma concentrations of palmitoleic, linoleic, palmitic, oleic, and stearic acids 24 h after 20, 40, and 80 mg/kg oleic acid doses (mean ± SEM of 5 different experiments). (b) Total NEFA concentration (sum of average concentrations of the five NEFA). All mice receiving the 160 mg/kg dose died 10–20 minutes after the intravenous injection and could not be computed. (c) Plasma concentrations of palmitoleic, linoleic, palmitic, oleic, and stearic acids 6 h after the injection of the 80 mg/kg oleic acid dose (mean ± SEM of 4 independent experiments). *P < 0.05 (versus controls).

 When mice were intravenously injected with increasing OLA doses (20, 40 and 80 mg/kg), a dose-dependent decrease in plasma NEFA concentrations especially oleic, linoleic and palmitic acids (Figure 1(a)), and total fatty acids (Figure 1(b)) were observed 24 h after the injection. To define if this effect could be detected at an earlier moment, we performed an experiment evaluating NEFA concentrations 6 h after OLA injection, using the 80 mg/kg OLA dose (this dose corresponded to the maximal response obtained in the experiment of Figure 1(a)). Results for this early-time point (Figure 1(c)) showed only minor decreases relative to controls in some NEFA concentrations which were not statistically significant. Albumin levels were only slightly altered (Table 1). 

**Figure 2:**
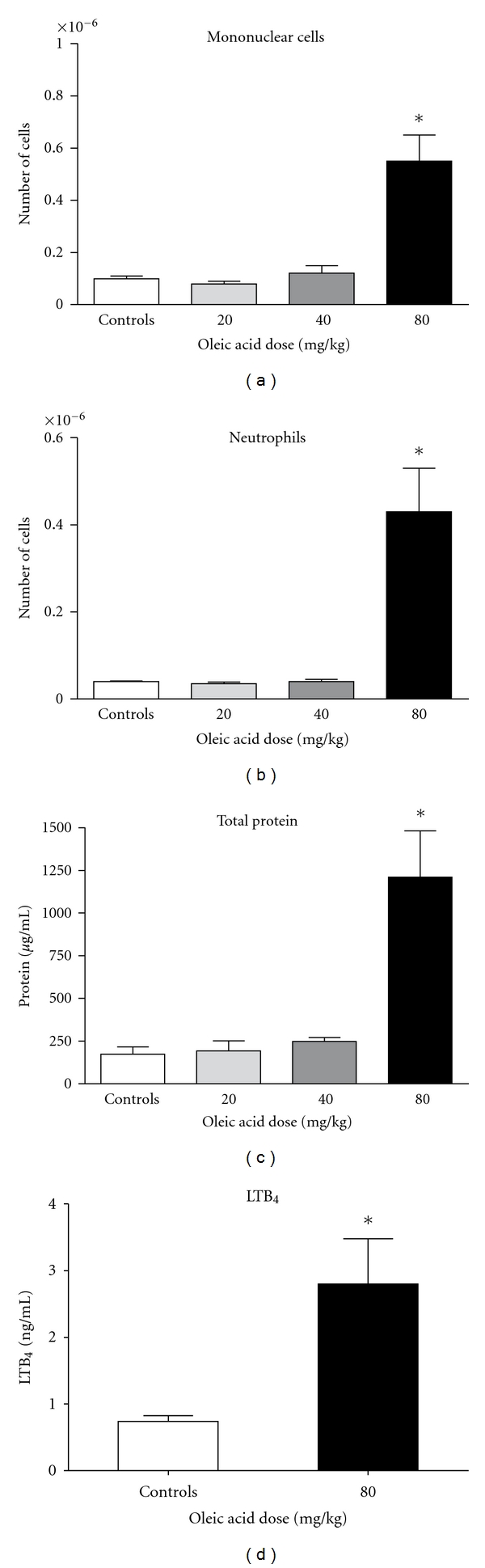
Leukocyte migration, protein extravasation, and LTB_4_ production in BALF after intravenous OLA injections. Mononuclear cells, neutrophils, and total protein (in BALF supernatants) were measured 24 h after injections, while LTB_4_ was assayed in supernatants 6 h after injections. Results represent the mean ± SEM of 3 independent experiments. *P < 0.05.

**Figure 3:**
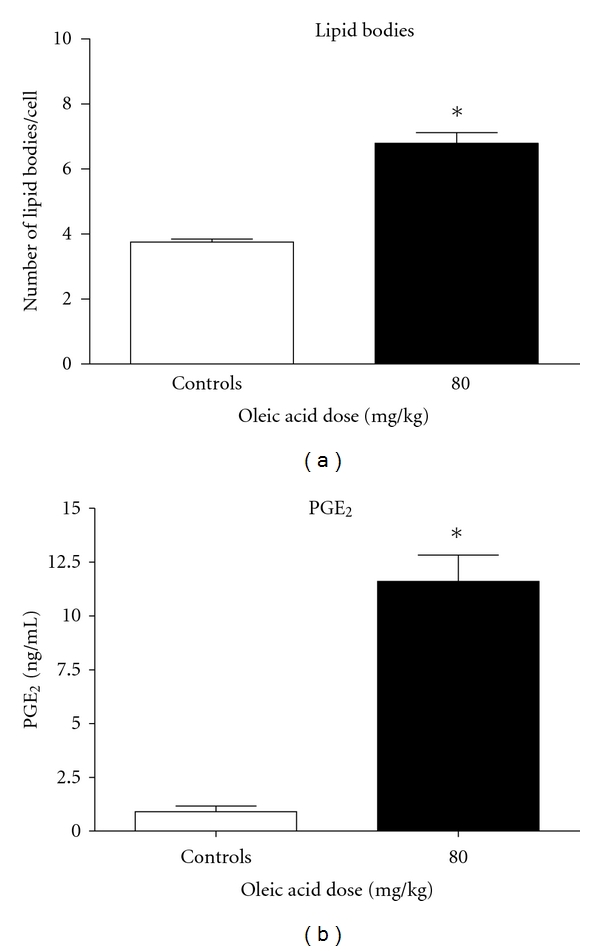
Lipid body formation and prostaglandin production in BALF after intravenous OLA injections. BALF were collected 24 h after injections of the 80 mg/kg dose. Each bar represents the mean ± SEM of lipid bodies per cell in 50 consecutively counted cells from 6 different animals. PGE_2_ quantification is the mean ± SEM of 3 independent experiments. *P < 0.05.

 In order to characterize the onset of ALI, we measured in BALF samples the following parameters: protein extravasation, leukocyte accumulation, lipid body formation in leukocytes and PGE_2_ and LTB_4_ production, which were used as markers of lung edema and inflammation. Although OLA is potentially able to induce lung injury, intravenously injected OLA in 20 and 40 mg/kg doses did not induce BALF cell migration or did not produce modifications on protein BALF content (Figure 2). Notwithstanding, 24 h after the 80 mg/kg dose, an infiltration of mononuclear cells and neutrophils, as well as an augmented total BALF protein, was detected. LTB_4_ was also significantly increased 6 h after this challenge (Figure 2). Lipid bodies in BALF leukocytes and the lipid mediator PGE_2_ in BALF supernatant (Figure 3) were also considerably augmented 24 h after this same OLA challenge. A dose of 160 mg/kg killed all mice. These animals presented early signs of severe lung injury and died within 10 minutes after injections.

**Figure 4:**
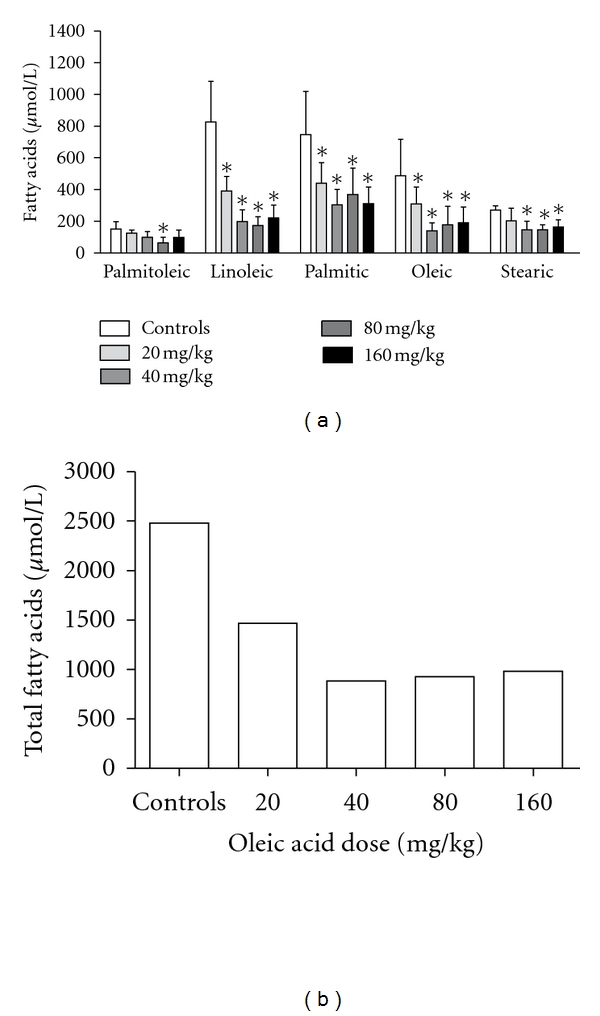
Plasma NEFA concentrations after OLA administration by the gastric route. (a) Plasma concentrations of palmitoleic, linoleic, palmitic, oleic, and stearic acids 24 h after 20, 40, 80, and 160 mg/kg oleic acid doses; (b) total NEFA concentration (sum of average concentrations of the five NEFA). Results represent data from 5 independent experiments (mean ± SEM). *P < 0.05.

**Figure 5:**
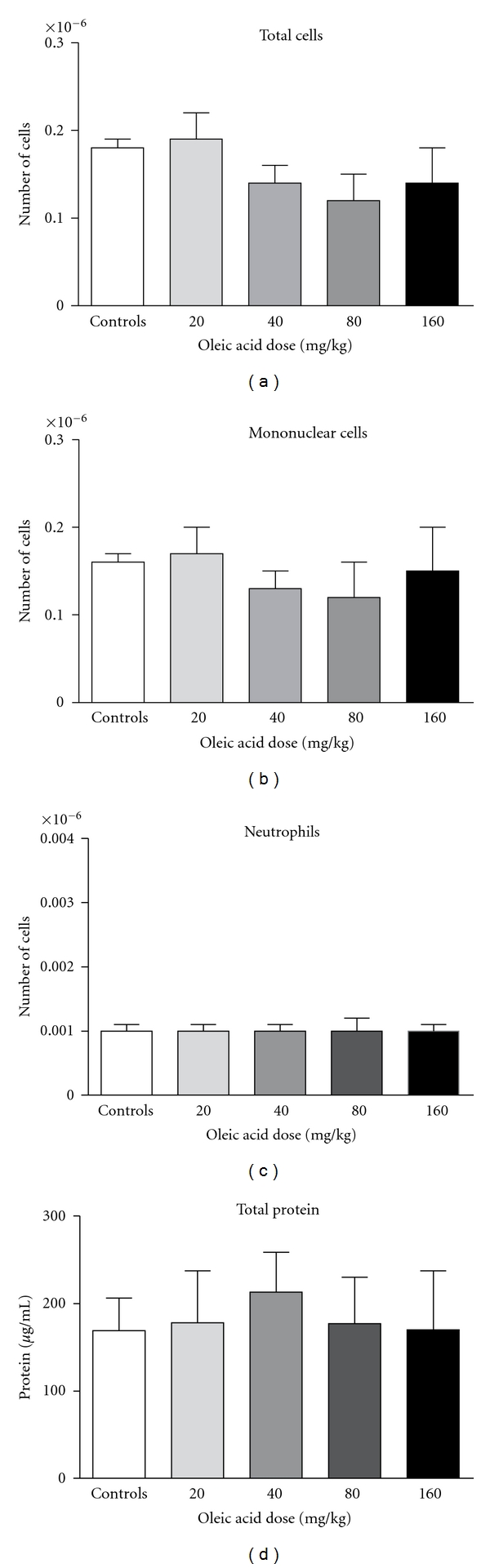
Leukocyte migration and protein extravasation in BALF after OLA administration by the gastric route. Bronchoalveolar fluids were collected 24 h after OLA administration. Total cells, mononuclear cells, and neutrophils were counted. Total protein in BALF supernatants was also assayed. Results represent the mean ± SEM of 3 independent experiments.

When the same OLA doses were administered to mice by the enteral route, lowered individual and total NEFA concentrations were also detected (Figures 4(a) and 4(b), resp.). This decrease was substantially more pronounced than the one seen in intravenously treated animals. Lung injury was not found even in the highest dose tested as can be seen in Figure 5. Since lung edema and leukocyte infiltration were not detected in this experiment, assays for inflammatory mediators were not performed.

## 4. Discussion

Herein we demonstrated an unexpected decrease in NEFA plasma levels after intravenous or enteral OLA administration. In this regard, several studies have shown that fatty acids can regulate its own metabolism, acting at gene transcription level. Some transcription factors are prospective fatty acid targets regulating the expression of enzymes involved in lipid metabolism [35–38]. Nonesterified fatty acid availability is sensed by peroxisome proliferator-activated receptors (PPARs), which are nuclear receptors controlling fatty acid storage, degradation, and adipocyte differentiation [39, 40]. Although in the present study we did not test OLA binding to PPAR, this fatty acid was already reported so effective as polyunsaturated fatty acids in PPAR*α* binding and activation [41] and was also a PPAR*γ* ligand [42]. In fact, PPAR*α* activation in the liver stimulates the transcription of carnitine palmitoil-transferase 1 (CPT1) and uncoupling protein 1, leading to increased fatty acid degradation [43]. Fish oils contain PPAR*α* activators that, similarly to hypolipidemic drugs, decreased triglyceride synthesis and increased mitochondrial fatty acid *β*-oxidation [44]. PPAR*γ* activation augmented fatty acid clearance by the adipose tissue and hepatocytes, consequently decreasing their plasma concentrations [45]. Hence, PPAR activation seems to be an important condition decreasing nonesterified fatty acid blood concentrations. In this way, PPAR*γ* agonists lowered plasmatic NEFA concentration [45] while PPAR*α* agonists led to a similar effect by increasing NEFA oxidation [46].

Mice receiving OLA through the intravenous route (80 mg/kg) already presented signals of lung injury, characterized by increased protein extravasation, cell migration and cell activation with increased lipid body formation and PGE_2_ release. Moreover, LTB_4_, a potent neutrophil chemotactic molecule [47], was augmented at an early stage, thus contributing for neutrophil migration. In our conditions, OLA lung toxicity can be explained by the rapid arrival of albumin unbound-OLA in the lung capillary net. It is important to note that this amount of OLA, if diluted in the whole mouse blood (considered as 2.5 mL), would give a concentration of at least 4000 *μ*mol/L, which is around 1.7 and 6.6 times the control levels of total fatty acid and OLA, respectively. Surely, during the few seconds of traveling from the injection point to lung, OLA concentration would be much higher than 4000 *μ*mol/L. Moreover, a 160 mg/kg intravenous dose killed all animals within 10 min after injections, a toxic effect certainly due to albumin unbound-OLA. In this context, it was proposed that the toxicity of intravenously administered OLA could be diminished by a concomitant albumin injection [48].

OLA enteral administration was not toxic in any tested doses. Since an appreciable part of OLA undergoes esterification during the intestinal absorptive mechanism and considering that intestinal absorption is a much slower process, an increase in albumin unbound-OLA is prevented in this condition. It is worth of note that OLA administration by the gastric route (40–80 mg/kg) was twice as much efficient in lowering total plasma NEFA (a decrease of about 60%) than the intravenous administration (around 30%). At this point, we would like to emphasize published data showing that mice consuming olive oil-enriched diet (thus an OLA enriched-diet) had increased survival after a LPS induced-shock [49]. This shock is characteristically seen in sepsis, a disease coursing with high plasma NEFA concentrations. In this case, diet-induced-reduced-plasma NEFA could be an explanation for the extended mice survival.

There are evident differences in OLA distribution in the body when this fatty acid is administered by intravenous or enteral routes. In intravenously treated animals, OLA is rapidly and significantly trapped in the lung microvasculature causing lung inflammation. After enteral administration, OLA is mostly esterified and transported through the abdominal lymphatic system then reaching the venous system, heart, lung and, afterwards, is distributed to the whole organism. The enteral route follows, thus, the normal physiologic mechanisms of lipid absorption and transport.

Other nonesterified fatty acids may have similar effects on NEFA plasma levels. In this work, OLA was chosen because not only toxic but also benefic effects of this fatty acid are well documented in the literature.

## 5. Conclusions

In conclusion, OLA seems to participate in the regulation of fatty acid metabolism. Intravenous OLA administration (40 mg per kg of body weight) lowered plasma NEFA concentrations, but higher doses were toxic, leading to lung injury or killing the animals. On the other side, our results suggest a benefic effect of low doses of orally administered OLA (about 40 to 80 mg per kg of body weight) in reducing plasma NEFA concentrations of normal animals. This finding sum up the other benefits brought about by the ingestion of diets containing OLA-enriched fat, particularly olive oil.

## Figures and Tables

**Table 1:** Plasma albumin concentrations 24 h after intravenous (I.V.) and intragastric (I.G.) OLA administration.

		OLA dose (mg/kg)			
	Controls	20	40	80	160
Albumin (*μ*M) I.V. OLA	357.2 ± 8.1	338.6 ± 8.8	325±20.0	301.6 ± 18.2	nd
Albumin (*μ*M) I.G. OLA	367.3±12.1	333.3 ± 9.6	326.4 ± 19.1	319.0 ± 34.1	350.1 ± 19.4

Nd: not determined.

Results are mean ± SEM of 6 to 7 different experiments.

## Abbreviations

ALI: Acute lung injury
ARDS: Acute respiratory distress syndrome
BALF: Bronchoalveolar lavage fluid
CPT1: Carnitine palmitoil-transferase 1
NEFA: Nonesterified fatty acids
HPLC: High performance liquid chromatography
IL: Interleukin
LTB_4_: Leukotriene B_4_
IV: Intravenous
IG: Intragastric
LNA: Linoleic acid (18 : 2n-6)
OLA: Oleic acid (18 : 1n-9)
PGE_2_: Prostaglandin E_2_
PPAR: Peroxisome proliferator-activated receptors
TNF: Tumor necrosis factor.
